# Innate lymphoid cells in immunoglobulin E-mediated food allergy

**DOI:** 10.1097/ACI.0000000000001018

**Published:** 2024-08-12

**Authors:** Janarthanan Ilangovan, Joana F. Neves, Alexandra F. Santos

**Affiliations:** a Peter Gorer Department of Immunobiology, School of Immunology and Microbial Sciences, Faculty of Life Sciences and Medicine; b Centre for Host Microbiome Interactions; c Department of Women and Children's Health (Paediatric Allergy), School of Life Course Sciences, Faculty of Life Sciences and Medicine, King's College London; d Children's Allergy Service, Guy's and St Thomas’ Hospital, London, UK

**Keywords:** allergen, anaphylaxis, food allergy, immunoglobulin E, innate lymphoid cells, oral tolerance

## Abstract

**Purpose of review:**

Recognition of the importance of innate lymphoid cells (ILCs) in the immune mechanisms of food allergy has grown in recent years. This review summarizes recent findings of ILCs in immunoglobulin E (IgE)-mediated food allergy. New research on ILCs in the context of the microbiome and other atopic diseases are also considered with respect to how they can inform understanding of the role of ILCs in food allergy.

**Recent findings:**

ILCs can mediate allergic and tolerogenic responses through multiple pathways. A novel subset of interleukin (IL)-10 producing ILC2s are associated with tolerance following immunotherapy to grass pollen, house dust mite allergy and lipid transfer protein allergy. ILC2s can drive food allergen-specific T cell responses in an antigen-specific manner. A memory subset of ILC2s has been identified through studies of other atopic diseases and is associated with effectiveness of response to therapy.

**Summary:**

The role of ILCs in food allergy and oral tolerance is relatively understudied compared to other diseases. ILCs can modulate immune responses through several mechanisms, and it is likely that these are of importance in the context of food allergy. Better understanding of theses pathways may help to answer fundamental questions regarding the development of food allergy and lead to novel therapeutic targets and treatment.

## INTRODUCTION

Two million people in the UK are estimated to have a diagnosed food allergy and evidence suggests that the prevalence of this condition is rising throughout the western world [1,2]. Defined as an adverse immunological response to a food protein, there is no curative treatment available. The standard approach of food avoidance poses many risks for the patient and has a significant social, physical and economic impact [3].

Oral immunotherapy (OIT) is the only approved treatment for peanut allergy in the UK and while it is able to raise the threshold of response, adverse reactions during treatment can occur and the majority of patients are unable to achieve sustained unresponsiveness beyond treatment [4]. This highlights a clear need for a better understanding of the underlying mechanisms that define the allergic response to aid in the development of curative treatments.

Food allergy is driven by a dysregulated type 2 immune response and following their relatively recent discovery there is growing appreciation of the importance of innate lymphoid cells (ILCs) in defining this response. This review summarizes recent findings of ILCs in immunoglobulin E (IgE)-mediated food allergy and reflect on how studies of ILCs in other atopic conditions may offer insight into their role in food allergy. 

**Box 1:**
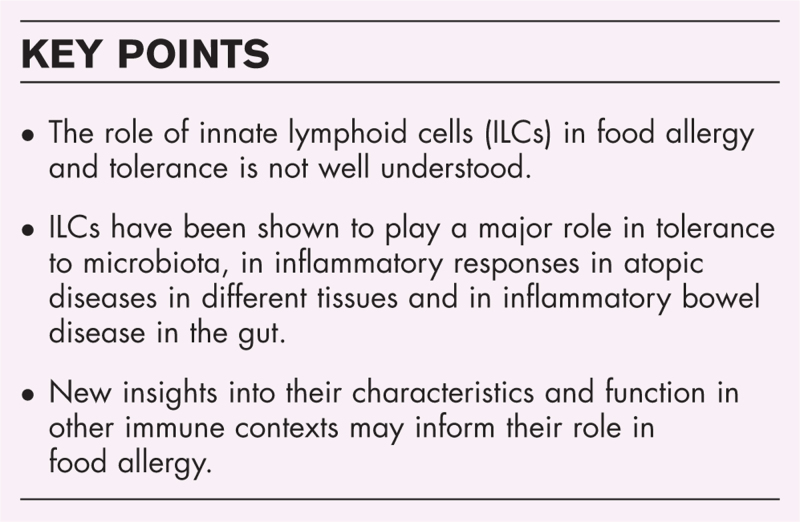
no caption available

### The food allergic immune response

Food allergy can be divided into IgE-mediated, mixed IgE and non-IgE mediated (e.g. eosinophilic esophagitis) and non-IgE mediated responses (e.g. food protein-induced enterocolitis syndrome). The focus of this review is IgE-mediated food allergy, which is characterized by the presence of allergen-specific IgE antibodies.

The immune response following initial allergic sensitization to foods has been studied extensively. Food antigens are transported across the intestinal epithelium and presented by CD103^+^ dendritic cells (DCs) to naïve T cells in the gut associated lymphoid tissue. In tolerance, this induces polarization towards FoxP3+ regulatory T cells (Tregs) in a retinoic acid and TGF-β-dependent manner [5]. Tregs support tolerance in many ways including suppression of IgE via preferential IgA and IgG production, alteration of mast-cell activity and inhibition of the Th2 response [6–8]. Dysfunction of tolerance, instead, results in induction of T helper type 2 (TH2) cells and T follicular helper (Tfh) 13 cells. TH2 cells increase chemoattraction of eosinophils and production of mast cells while inhibiting Treg production [9]. Tfh cells, specifically the subset Tfh13, induce the production of high-affinity, allergen-specific IgE via B cell class switching which binds with high-affinity to FcεRI receptors on the surface of mast cells and basophils [10]. Subsequent exposure to the allergen leads to crosslinking of receptor bound IgEs, activation and degranulation of mast cells and basophils with release of mediators that further drive a pro-inflammatory response.

While there is no consensus on the factors that lead to the development of food allergy, research by Lack *et al.* has shown that early introduction of food allergens dramatically reduces the likelihood of developing food allergy in high-risk children [11]. Recently, the follow up LEAP trio study demonstrated that this reduction is sustained beyond early childhood regardless of follow up peanut consumption, providing firm evidence that early oral exposure to food antigens induces long-term tolerance [12^▪^]. Hence, while the precise mechanism underlying this induction of tolerance is not fully understood, these findings suggest that key players likely exist in the gut.

### Innate lymphoid cells

ILCs are a subtype of lymphocytes belonging to the innate immune system. They are divided into five subsets according to their transcription factor expression, cytokine production and function: natural killer cells, lymphoid tissue inducer cells and the helper-like subsets ILC1, ILC2, and ILC3. ILCs lack antigen-specific receptors and, instead, are activated by the cytokine milieu of their microenvironment and a range of other signalling molecules such as microbial peptides and neuronal transmitters. ILC1s can be regulated by the transcription factor T-bet, activated by interleukin (IL)-12, IL-18 and produce interferon (IFN)-γ and TGF-β1. ILC2s are transcriptionally regulated by the GATA 3 transcription factor and retinoic acid receptor-related orphan receptor α (RORα) and are capable of producing IL-4, IL-5 and IL-13 upon activation by the alarmins IL-25, IL-33 and thymic stromal lymphopoietin (TSLP). ILC3s are characterized by expression of retinoic acid-related orphan receptor transcription factor (RORyT). They are activated by IL-1β and IL-23, leading to the secretion of IL-17, IL-22, granulocyte-macrophage colony-stimulating factor (GM-CSF) and tumour necrosis factor (TNF)-α. ILC3s can be further divided based on expression of the natural cytotoxicity receptor NKp44 [13]. Of particular interest with regards to food allergy are the ILC2s and ILC3 subsets, which can be considered the innate counterparts of CD4^+^ Th2 and Th17/Th22 cells respectively.

ILCs show considerable heterogeneity across and within tissues and plasticity between different ILC subtypes have been found in response to microenvironmental changes [14,15]. This allows for a refined and flexible immune response that can be mounted rapidly, independent of antigen specificity. Differentiation between subsets has been associated with the immune response to infection and chronic inflammation but it is not clear what role their plasticity has in the context of food allergy. For example, it was recently found that the traditional ILC2 marker prostaglandin DP2 receptor (CRTH2) is not expressed in a subset of ILC2s associated with severe asthma [16]. In part due to this plasticity, there is inconsistency across the literature on the appropriate way to define ILCs. This has made deciphering the characteristics and functions of ILCs within each subset more difficult. An additional challenge to studying ILCs in humans is their accessibility. ILCs are mainly tissue resident, and represent a rare population in circulation, unlike their T helper cell counterparts. While reports show that ILCs at intestinal and skin barrier sites can migrate to distant tissues to support local immune responses, access to mature ILCs in peripheral blood remains limited [17,18].

### Innate lymphoid cells in food allergy

Overall research into how ILCs are directly involved in food allergy is lacking compared to other atopic conditions, nevertheless, there have been a number of studies demonstrating their importance in mediating the food allergic responses.

ILC2s, as potent producers of type 2 cytokines, have been strongly implicated in promoting the pro-inflammatory response in food allergy. This has been shown to occur through inhibition of Tregs by ILC2s via IL-4 and promotion of mast cell activation in an experimental model of food allergy where expansion of ILC2s was IL-33 dependent [19]. IgE-mediated activation of mast cells also triggers ILC2 to produce of IL-13 which in turn increases tissue sensitivity to mast cell mediators, thereby exacerbating the severity of anaphylaxis [20]. In another murine model of peanut allergy induced by airway sensitization, ILC2s promoted Tfh cell development and consequent IgE to peanut in an IL-13 dependent manner [21]. ILC2s have also been shown to drive mast-cell expansion in an IL-4 dependent manner leading to promotion of anaphylaxis likely through increased intestinal permeability [22]. In contrast, ILC2s have a role in intestinal homeostasis by promoting IL-13 dependent renewal of epithelial stem cell as well as the differentiation of mucus-producing goblet cells which protect against helminth infections [23,24]. Following injury, ILC2 can support tissue repair through the production of the growth factor amphiregulin which is produced by ILC2s in an IL-33 dependent manner [25].

Less is known about the role of ILC3s in food allergy, but they have been extensively associated with promoting homeostasis in the gut, where tolerance begins. For example, through IL-22 production they play a key role in maintenance of intestinal barrier integrity [26]. ILC3s can aid in the development of tolerance to oral antigens through multiple mechanisms, driving differentiation of Tregs through IL-2 production and promoting Tregs indirectly through GM-CSF dependent cross-talk with macrophages and dendritic cells [27,28].

### Innate lymphoid cells and the gut microbiota in food allergy

Communication between the gut microbiome and the immune system is essential for intestinal homeostasis and there are numerous examples of perturbation leading to disease. In humans, specific microbial species have been associated with supporting tolerance or development of allergy and research has shown that transfer of microbiota from healthy but not food allergic infants is protective against anaphylaxis [29,30].

Short chain fatty acids (SCFA) produced by fermentation of dietary fibres are one of the major microbiota metabolites associated with promoting tolerance [31]. This occurs largely through their ability to drive expansion of Tregs via multiple pathways. In the context of ILCs, SCFA are important for their proliferation in the intestine during development and help support intestinal homeostasis by ILC3-mediated IL-22 production [32,33]. Tryptophan metabolites produced by the gut microbiota similarly support homeostasis through ILC3-derived IL-22 production, this time through binding of aryl hydrocarbon receptor on ILC3s [34].

Presentation of microbial peptides is an essential step in the maintenance of tolerance to commensal bacteria in the gut. It was traditionally believed that conventional antigen presenting cells such as DCs were the major contributor to this tolerance. However, over the past 10 years, there has been increasing recognition of the ability of ILC3s to mediate tolerance through antigen presentation. This interest culminated in a series of recent studies which have shown that RORyt+ cells, including ILC3 s, are both necessary and sufficient to maintain tolerance against commensal bacteria [35^▪▪^,^36^–^38^]. Tolerance occurs through suppression of Th17 polarization of naïve T cells and expansion of microbiota specific Tregs in an MHCII-dependent manner aided by αVβ3 integrin that processes latent TGFβ [35^▪▪^]. In a separate report, it was shown that ILCs regulated tolerance to commensal bacteria via suppression of Tfh responses and B-cell class switching [39]. Despite the parallels between the pathways described here and development of tolerance to food antigens there is limited evidence of these mechanisms in food allergy. A recent study noted an increase in RORyt+ MHCII+ ILC3s following induction of tolerance to ovalbumin [40^▪^]. Interestingly, this expansion occurred independently of changes in the gut microbiota suggesting that nonmicrobial signals such as food antigens are able to regulate this cell population. The studies described here were performed in mouse models but, promisingly, these ILC3s with antigen-presenting capabilities have been identified in humans [41].

Less is known about potential ILC2 MHCII-dependent mechanisms. MHCII+ ILC2 s were first described in a mouse model in mediating helminth expulsion, and since then functional antigen-presenting ILC2 s which can drive T cell responses have been reported in humans following exposure to the inflammasome associated cytokines IL-1β and IL-18 [41,42]. Of note, it was also recently described that a similar MHCII+ ILC2 subset was capable of driving antigen-specific T cell response to the lipid transfer protein (LTP) allergen, Pru-P-3 [43]. However, further investigation is needed to understand the importance of MHCII-dependent ILC function in food allergy.

### Lessons from other atopic diseases

Since their discovery, understanding of the role ILCs play in other atopic diseases has improved dramatically and it is likely that some of these mechanisms are conserved in food allergy.

One example is the discovery of ‘regulatory ILC2s’ first identified in a mouse model of asthma which produce the anti-inflammatory cytokine IL-10 although without expression of the classical regulatory T cell transcription factor FOXP3 [44]. This subset has been noted in multiple atopic diseases such as their presence in the nasal polyp tissue of patients with chronic rhinosinusitis [45]. Recent reports have demonstrated that a decrease in these IL-10 producing ILC2s is associated with house dust-mite allergic rhinitis and grass pollen allergy [46,47]. Furthermore, this population was then restored in patients who received immunotherapy. Following these findings, Palomares *et al.* demonstrated their importance in the context of food allergy where, similarly, IL-10-producing ILC2 s increased following Pru p 3 sublingual immunotherapy for the treatment of LTP allergy [48]. In a mouse model, ILC2s were shown to be the predominant source of IL-10 in the intestine [49]. It is unclear how long-lasting this population is, and its importance in the increasing threshold to reaction associated with immunotherapy, but nonetheless the role of this subset is of significant interest.

Another exciting new area of ILC research is the discovery of innate ‘memory’ cells in mice which can respond more strongly following previous stimulation. These ILC2 s proliferated and secreted typical type 2 cytokines in response to allergen or the alarmin IL-33 [50]. Following an expansion and contraction phase, a subset of these cells persisted after challenge and upon subsequent exposure to unrelated allergens produced a more severe type 2 response. In humans, a similar phenotype was described characterised by expression of the marker CD45RO on ILC2s. Termed as ‘inflammatory ILC2s’, these are derived from CD45RA+ naïve ILCs following alarmin stimulation and, interestingly, were found to be increased in peripheral blood of patients with chronic rhinosinusitis or asthma [51]. Furthermore, their presence correlated with disease severity and resistance to steroid treatment. Very recently, it was confirmed that these inflammatory ILC2s do, in fact, have the same properties as the memory ILC2s in mice [52^▪▪^]. In response to activation these cells also downregulated CD127, the conventional ILC marker, in part explaining why they have remained undiscovered until now.

Recent research has demonstrated the clinical relevance of this inflammatory/memory subset of ILCs as their presence is associated with faster response to the drug dupilumab [53^▪^]. Dupilumab blocks the IL-4/IL-13 alpha receptor, which is a target of Th2 cytokines extensively produced by inflammatory ILC2s. The existence of this memory population in food allergy has not been described but has important possible ramifications. It could, for instance, represent a potential biomarker of treatment response, as it was recently shown that dupilumab significantly decreases specific IgE to multiple food allergens, although these patients were not assessed by oral food challenge [54^▪^]. It may also explain why almost 40% of patients with food allergy have multiple food allergies since this inflammatory profile is non antigen-specific [55,56].

In mice, a similar inflammatory ILC3 population has been identified responding to bacterial infection in the gut. [57]. Described as ‘trained’ ILC3s, they were capable of increasing production of IL-22, better controlling infection and responding nonspecifically to other bacterial species. So far, these cells have not been identified in humans nor associated with food allergy but are of interest for their potential role in mediating tolerance.

## CONCLUSION AND FUTURE DIRECTIONS

**FIGURE 1:**
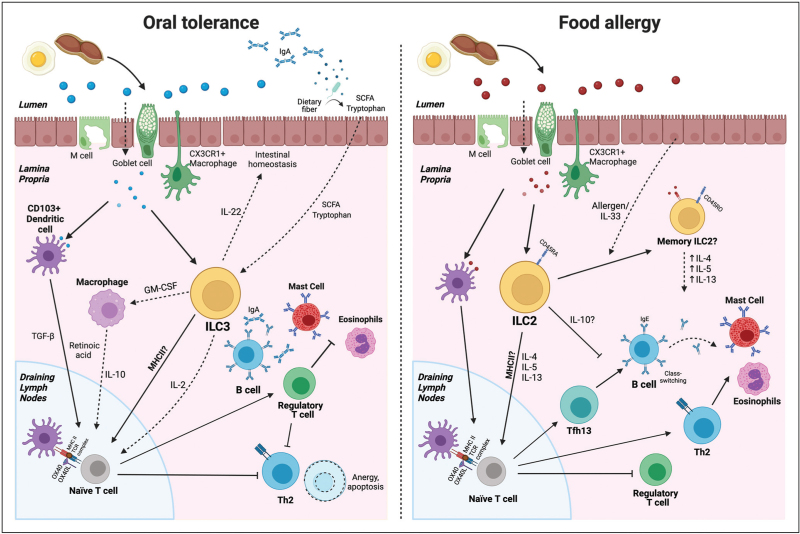
The known and potential role of ILCs in food allergy and tolerance – mechanisms through which ILCs are known to drive oral tolerance or food allergy and potential pathways based on findings from other atopic diseases. ILCs, innate lymphoid cells.

ILCs represent a relatively recent discovery in immunology research and our understanding of these cells lags behind many other elements of the immune system. In this review, we have highlighted several recent findings, summarized in Fig. 1, that have deepened our understanding of the role ILCs play in mediating allergic responses. ILCs represent a diverse subset of the immune system, and, as discussed here, they participate in several mechanisms underlying atopic diseases that are only just starting to be appreciated. Better understanding of how these contribute to the development of food allergy and oral tolerance is urgently needed. A number of biologics targeting ILC pathways, such as dupilumab (anti IL-4/IL-13 receptor), etokimab (anti-IL-33) and tepezulmab (anti-TSLP) have been trialled in food allergy and other atopic diseases [54^▪^,^58^,^59^]. As research progresses, these cells may provide insight into fundamental questions regarding the mechanisms behind the development of food allergy and maintenance of oral tolerance.

## Acknowledgements

*None.*

### Financial support and sponsorship

*J.I. is supported by the UK Medical Research Council (MR/N013700/1.) and is a King's College London member of the MRC Doctoral Training Partnership in Biomedical Sciences. J.F. Neves is funded by the Lister Institute of Preventive Medicine Prize, BBSRC and the NC3Rs (NC/X002497/1) and The Leona M. and Harry B. Helmsley Charitable Trust. A.F. Santos is funded by the Medical Research Council (MRC Transition support MR/T032081/1, MRC Clinician Scientist Fellowship MR/M008517/1, MRC Centenary Early Career Award and MRC Clinical Research Training Fellowship G0902018), the Immune Tolerance Network/National Institute of Allergy and Infectious Diseases (NIAID, NIH), Asthma UK (AUK-BC-2015-01), Food Allergy Research and Education, BBSRC, Rosetrees Trust and the NIHR through the Biomedical Research Centre (BRC) award to Guy's and St Thomas’ NHS Foundation Trust, in partnership with the King's College London and King's College Hospital NHS Foundation Trust.*

*Funding: This work was supported by the Medical Research Council and the MRC-DTP PhD Programme at King's College London.*

### Conflicts of interest

*A.F.S. reports grants from Medical Research Council (MR/M008517/1; MC/PC/18052; MR/T032081/1), Food Allergy Research and Education (FARE), the Immune Tolerance Network/National Institute of Allergy and Infectious Diseases (NIAID, NIH), Asthma UK (AUK-BC-2015-01), BBSRC, Rosetrees Trust and the NIHR through the Biomedical Research Centre (BRC) award to Guy's and St Thomas’ NHS Foundation Trust, during the conduct of the study; personal fees from Thermo Scientific, Nestle, Nutricia, Infomed, Novartis, Allergy Therapeutics, IgGenix, Buhlmann, as well as research support from IgGenix, Buhlmann and Thermo Fisher Scientific through a collaboration agreement with King's College London. The other authors have nothing to disclose.*

## REFERENCES AND RECOMMENDED READING

Papers of particular interest, published within the annual period of review, have been highlighted as:

▪ of special interest
▪▪ of outstanding interest
